# Treatment compliance, treatment patterns, and healthcare utilization in epilepsy patients with first add-on antiepileptic drugs: A nationwide cohort study

**DOI:** 10.1016/j.heliyon.2024.e27770

**Published:** 2024-03-07

**Authors:** Min Young Kim, Jung-Ae Kim, Youngeun Lee, Sang Kun Lee

**Affiliations:** a Medical, Eisai Korea Inc, 6 Bongeunsa-ro 86-gil, Gangnam-gu, Seoul, Republic of Korea; b Real World Insights, IQVIA Korea, 173 Toegye-ro, Jung-gu, Seoul, Republic of Korea; c Department of Neurology, Seoul National University Hospital, 101 Daehak-ro, Jongno-gu, Seoul, Republic of Korea

**Keywords:** Anti-epilepsy drug, Epilepsy, Add-on therapy, Persistence, Adherence, Healthcare resource utilization

## Abstract

**Objective:**

This study aimed to assess the treatment compliance, patterns, healthcare resource utilization (HCRU), and costs of anti-epilepsy drugs (AEDs) as the first add-on therapy in patients with epilepsy.

**Methods:**

We conducted a retrospective population-based cohort study using Korean National Health Insurance claims data from 2016 to 2020. Patients with epilepsy who newly received AED add-on therapy were identified and followed for up to 12 months to evaluate persistence, adherence, treatment patterns, HCRU, and costs.

**Results:**

Among 6,746 patients who initiated AED add-on therapy, 65.5% were persistent on their index AED add-on from the index date until the end of the follow-up period, and the mean persistent time on the index add-on was 307.3 ± 92.3 days. A total of 76.8% patients were adherent, with a medication possession ratio (MPR) ≥80%, and the mean MPR was 88.9 ± 25.4%. Persistence and adherence to the index AED add-on were relatively higher among patients prescribed lamotrigine, levetiracetam, oxcarbazepine, and perampanel than those prescribed carbamazepine, topiramate, or valproate. A total of 41.0% of the patients changed their index AED add-on during the follow-up period. The carbamazepine, topiramate, and valproate groups had higher rates of change than the other AED groups. HCRU and costs tended to be lower in the lamotrigine, levetiracetam, oxcarbazepine, and perampanel groups. Furthermore, perampanel showed the lowest HCRU and costs for all-cause cases as well as the lowest length of stay and outpatient visits for epilepsy-related cases.

**Conclusion:**

In this population-based study, the use of lamotrigine, levetiracetam, oxcarbazepine, or perampanel as the first add-on therapy in patients with epilepsy contributed to better treatment compliance and lower HCRU and costs than that of carbamazepine, topiramate, or valproate.

## 1 Introduction

Epilepsy is one of the most common chronic neurological disorders which affects approximately 45.9 million people worldwide and accounts for more than 0.5% of the global burden of disease [[1], [2], [3]]. Antiepileptic drugs (AEDs) are the mainstay of treatment for epilepsy, and monotherapy is recommended as the initial treatment for newly diagnosed patients [4]. However, over 30% of patients fail to achieve adequate seizure control using initial monotherapy [5]. In such cases, switching to a different monotherapy or adding another AED are considered as alternative treatment options [6,7].

Previous studies have suggested the potential benefits of add-on therapy for seizures that are not successfully controlled by monotherapy [[8], [9], [10]]. However, the World Health Organization identified that the use of combined AEDs and complex medication regimens were associated with non-adherence [11].

Poor adherence is reported to be the main cause of unsuccessful epilepsy management and directly associated with a higher risk of seizure recurrence, reduced productivity, decreased quality of life, and greater healthcare utilization and costs. According to a large population-based study, non-adherence to AED was associated with three-fold higher risk of mortality than adherence and significantly higher risk of clinical events, including hospitalization, emergency room (ED) visit, and fractures. Similarly, non-adherence was reported to have increased frequency of seizures than adherent [[12], [13], [14], [15]]. Furthermore, since AED treatment is recommended for at least 2 years to attain seizure-free outcomes, choosing optimal AED combination therapy that can synergistically control seizures with fewer adverse effects is essential [4,16]. Although adherence to add-on therapy is an important factor to successful epilepsy treatment, the real-word evidence regarding adherence to add-on AEDs remains limited.

In our previous study, we found that the treatment compliance to AEDs as the first add-on therapy was different across the index AED add-on therapies in epilepsy patients [17]. Based on this finding, we aimed to corroborate the effectiveness of AEDs as the first add-on therapy for epilepsy, measured by treatment compliance and healthcare utilization, with an extended assessment period for the initial monotherapy use. Additionally, we investigated the treatment patterns of switching, stopping, and augmentation in patients who discontinued their first add-on therapy. Our study considered carbamazepine (CBZ), lamotrigine (LTG), levetiracetam (LEV), oxcarbazepine (OXC), perampanel (PER), topiramate (TPM), and valproate (VAL) as the target AEDs which are the most prescribed add-on therapies in Korea [18].

## 2 Methods

### 2.1 Data source

This retrospective, nationwide population-based study was conducted utilizing the Korean National Health Insurance claims data from the Health Insurance Review and Assessment (HIRA) from January 2016 to December 2020. The HIRA claims database contains demographic and medical claims information including diagnosis, procedures, treatments, prescribed medications, and inpatient and outpatient care for more than 50 million South Koreans. South Korea has a single payer, universal, and compulsory health coverage program that covers approximately 98% of the population [19]. Therefore, the HIRA claims data represent almost the entire population and current real-world clinical practice in South Korea. Diagnoses were coded using the Korean Standard Classification of Diseases, 7th Revision (KCD-7) codes which is a modification of the International Classification of Diseases, 10th Revision (ICD-10), Clinical Modification codes. Medications and treatments including surgical procedures were coded using the HIRA reimbursed drug and procedure codes. This study was approved by the Institutional Review Board of Seoul National University Hospital (E−1811-002-982) and HIRA Data Review Committee (M20210226130). The informed consent was waived since the HIRA claims data was structured in de-identified format.

### 2.2 Study population

**Fig. 1:**
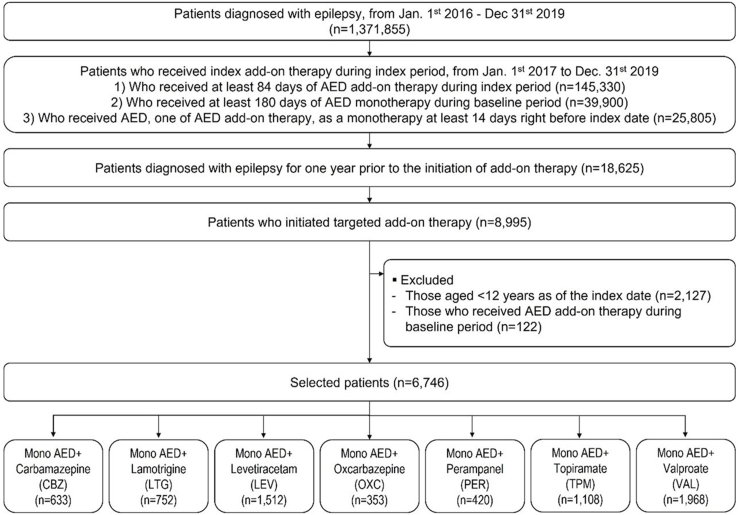
Flowchart of study population selection. AED, anti-epileptic drug; Mono, monotherapy.

Among patients who had ≥1 claims with the relevant ICD-10 codes (G40 or G41) for epilepsy as any diagnosis between January 2016 and December 2019, those who received one of the target AEDs overlapping with monotherapy for ≥84 days (including gap days of 15 days) from the index date, and those who were treated with monotherapy for ≥14 days immediately before the index date were included as in our former study [17]. In the present study, we included those who were on monotherapy for ≥180 days during the 12 months prior to the index date (i.e., baseline period) to further homogenize the study population. Patients aged <12 years were excluded. To enroll only those newly treated with AED add-on to monotherapy, we excluded patients who received AED add-on therapy during the baseline period were excluded. The selected patients were followed for up to 12 months from the index date to evaluate treatment compliance, treatment patterns, healthcare resource utilization (HCRU), and costs. Details of the patient inclusion/exclusion criteria and enrollment process are presented in Fig. 1 and Supplementary Table 1.

### 2.3 Study outcome

Treatment persistence was defined as the proportion of patients persistent on their index AED add-on therapy from the index date. Patients were considered persistent on their index AED add-on if they refilled their index AED add-on therapy within gap days, which was defined as a period between two consecutive prescriptions, of 50% of days' supply from the end of previous index AED add-on therapy. Persistent time on treatment was defined as the duration from the initiation to discontinuation of the index AED add-on therapy within the follow-up period. Treatment patterns were assessed as “change” or “no change” based on the subsequent AEDs prescribed within the defined gap days. Change was further classified as “switch/stop” or “augmented”. Switch/stop was defined as renewal with a different AED, addition of another AED to the index AED add-on with <28 overlapping days, or no subsequent prescription of the index AED within the defined gap days. Augmented change was defined as the addition of another AED to the index add-on with ≥28 overlapping days. Adherence was measured using the medication possession ratio (MPR), which was calculated as the proportion of total days’ supply of the index AED add-on during the 12-months of follow-up period. Adherent was defined as patients with MPR ≥80% [20,21].

All-cause and epilepsy-related HCRU and costs were assessed within the 12 months of follow-up period from the index date. Epilepsy-related HCRU and costs were defined as claims with epilepsy diagnostic codes as the primary diagnosis. HCRU and costs were categorized according to medical service type, including hospitalization, outpatient visits, and ED visits. Cost was estimated as the sum of the medical costs covered by the government payer and the out-of-pocket costs paid by patients. The cost was converted from South Korean won (KRW) to USD using the annual average currency exchange rate in 2017 (1 USD = 1,178.59 KRW) [22]. Detailed definitions and relevant codes of healthcare utilization are presented in Supplementary Table 2.

### 2.4 Statistical analysis

Descriptive statistics are reported for all study outcomes, including patient demographics, comorbidities, and concomitant drug use during the baseline period. Detailed definitions and relevant comorbidity codes are presented in Supplementary Table 3. Continuous variables are summarized as mean and standard deviation (SD), and categorical variables are presented as frequency and proportion (%). The differences between the study groups were compared using ANOVA and Kruskal-Wallis test for continuous variables and a chi-square test for categorical variables. Cumulative persistence rates were assessed using Kaplan-Meier analysis and compared using the log-rank test. HCRU and costs were estimated for those who used each healthcare service. The study outcomes were further assessed according to the baseline characteristics. All statistical analyses were performed using SAS version 9.4 software (SAS Institute, Cary, North Carolina, USA) and the latest stable version of R (version 4.1.3, R Core Team (2022), Vienna, Austria). All statistical tests were conducted using two-sided tests with a significance level of 0.05.

## 3 Results

### 3.1 Baseline characteristics

A total of 6,746 patients who newly received AED as an add-on to monotherapy were eligible for this study: 9.4%, 11.1%, 22.4%, 5.2%, 6.2%, 16.4%, and 29.2% of patients received CBZ, LTG, LEV, OCX, PER, TPM, and VAL, respectively, as the first AED add-on therapy (Fig. 1). The mean age of the study population was 44.9±20.5 years and 52.1% was male. 51.0% of the patients had psychiatric comorbidities such as depression, anxiety disorders, bipolar disorders or schizophrenia. Patients in the CBZ and VAL groups were older, had higher modified Charlson Comorbidity Index (mCCI) scores, and had more psychiatric and neurological comorbidities than those in the LTG, LEV, OXC, TPM, or PER groups. In addition, the LTG, LEV, OXC, and PER groups received their first AED add-on from tertiary hospitals and had a lower proportion of patients with medical aid coverage than the CBZ, VAL, or TPM groups (Table 1).

**Table 1:** Demographic and clinical characteristics of study population.

	**Overall**	**Type of index AED**							***p*-value**
		**Mono** **+CBZ**	**Mono** **+LTG**	**Mono** **+LEV**	**Mono** **+OXC**	**Mono** **+PER**	**Mono** **+TPM**	**Mono** **+VAL**	
	**(N=6746)**	**(N=633)**	**(N=752)**	**(N=1512)**	**(N=353)**	**(N=420)**	**(N=1108)**	**(N=1968)**	
**Age**									
Years (mean ± SD)	44.9 ± 20.5	55.9 ± 18.8	39.4 ± 19.5	42.9 ± 20.3	42.5 ± 21.8	37.2 ± 16.4	42.6 ± 19.4	48.4 ± 20.7	<0.0001
12 to <65	79.9	63.7	87.1	81.9	80.5	93.8	84.7	75.0	<0.0001
65+	20.1	36.3	12.9	18.1	19.6	6.2	15.3	25.0	
**Sex**									
Male	52.1	51.8	44.3	54.1	55.5	54.5	45.8	56.1	<0.0001
Female	47.9	48.2	55.7	45.9	44.5	45.5	54.2	44.0	
**Insurance type**									
Medical aid	19.8	29.5	14.9	13.8	12.2	10.0	20.7	26.0	<0.0001
Health insurance	80.2	70.5	85.1	86.2	87.8	90.0	79.3	74.0	
**mCCI**									
Score (mean ± SD)	1.4 ± 1.9	1.9 ± 2.0	1.1 ± 1.7	1.4 ± 2.0	1.3 ± 1.8	0.8 ± 1.4	1.3 ± 1.7	1.6 ± 2.0	<0.0001
0	47.7	34.3	53.2	49.8	48.4	64.5	49.8	43.4	<0.0001
1	13.8	13.4	16.1	13.4	14.5	12.9	16.2	12.1	
2	17.0	19.8	13.8	16.0	17.9	13.3	15.2	19.6	
3	9.1	12.6	8.6	8.5	8.5	4.1	9.0	9.8	
4+	12.5	19.9	8.2	12.2	10.8	5.2	9.8	15.1	
**Psychiatric comorbidity**									
Any psychiatric comorbidity	51.0	65.7	50.8	31.2	28.9	26.4	60.4	65.5	<0.0001
Depression	32.7	41.4	32.6	16.4	17.3	14.5	42.1	43.9	<0.0001
Anxiety disorders	35.5	41.6	34.6	21.0	21.0	18.1	44.2	46.4	<0^.^0001
Bipolar disorder	19.1	17.9	28.3	5.9	4.5	3.1	26.5	27.9	<0.0001
Schizophrenia	11.5	14.9	11.3	2.5	3.4	0.2	19.5	16.9	<0.0001
**Neurological comorbidity**									
Mental retardation	6.1	9.0	3.3	3.7	4.8	2.1	7.8	8.1	<0.0001
**Hospital type**									
Clinic	10.9	21.2	6.3	5.0	6.0	1.7	13.4	15.2	<0.0001
Hospital	10.8	15.0	6.5	5.0	3.4	0.5	11.7	18.5	
General hospital	34.9	36.0	32.5	38.0	38.5	20.0	36.1	34.8	
Tertiary hospital	43.5	27.8	54.8	52.0	52.1	77.9	38.8	31.6	
**Number of baseline AEDs**									
Number of AEDs (mean ± SD)	1.5 ± 0.7	1.6 ± 0.8	1.7 ± 0.8	1.4 ± 0.7	1.6 ± 0.7	1.4 ± 0.7	1.5 ± 0.7	1.5 ± 0.7	<0.0001
1	60.0	52.3	46.3	66.5	53.5	71.2	65.3	58.3	<0.0001
2+	40.0	47.7	53.7	33.5	46.5	28.8	34.7	41.7	
**Types of epilepsy**									
Epilepsy with partial seizure	19.0	17.4	16.1	22.5	27.2	24.5	16.4	16.7	<0.0001
Epilepsy with generalized seizure	8.9	9.0	10.8	9.5	6.0	4.3	8.5	9.3	
Epilepsy with unspecified seizure	60.6	55.6	60.1	62.7	59.2	70.5	59.8	59.2	
Unknown	11.6	18.0	13.0	5.4	7.7	0.7	15.3	14.8	

AED, anti-epileptic drug; Mono, monotherapy; CBZ, carbamazepine; LTG, lamotrigine; LEV, levetiracetam; OXC, oxcarbazepine; PER, perampanel; TPM, topiramate; VAL, valproate; SD, standard deviation, mCCI, modified Charlson Comorbidity Index.

*P-value* was derived from individual chi-square test or Kruskal-Walis test.

Demographic and socioeconomic characteristics such as age, sex, insurance type, hospital type, and type of epilepsy were measured at the index date.

Comorbidities such as mCCI score, psychiatric comorbidities, neurological comorbidities, and number of AEDs were measured during the baseline period (1 year before the index date).

Data are presented as mean ± SD or percentages (%).

### 3.2 Treatment compliance

**Fig. 2:**
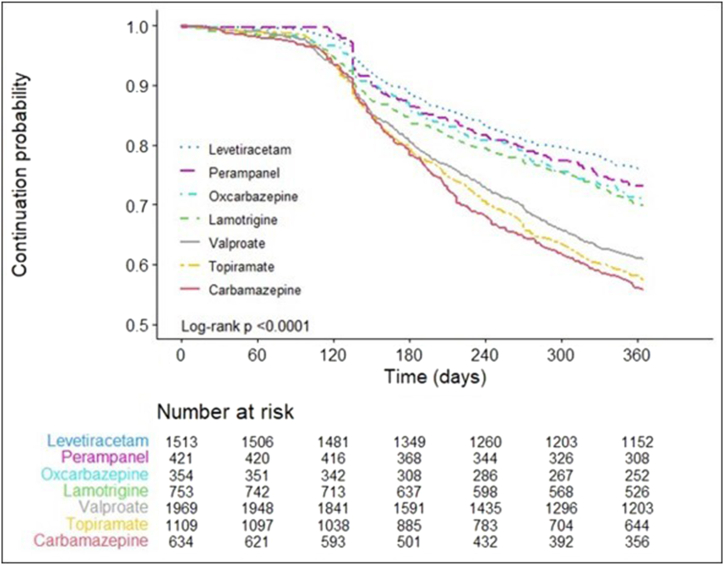
Kaplan-Meier curve for persistence by index anti-epileptic drug (AED).

In the entire study population, the mean persistence time on the index AED add-on to monotherapy was 307.3±92.3 days during the 12 months from the index date, and 4,420 (65.5%) patients were persistent on their index AED add-on at 365 days from the index date. The persistence time and rate were relatively higher among patients with LTG, LEV, OXC, and PER than those with CBZ, TPM, or VAL (Table 2, Supplementary Table 4). Furthermore, the LTG, LEV, OXC, and PER groups showed similar trends in persistence rate, whereas CBZ, TPM, and VAL groups showed similar one (Fig. 2).

**Table 2:** Persistence, adherence, and treatment pattern.

	Overall	Type of index AED							*p*-value
		Mono +CBZ	Mono +LTG	Mono +LEV	Mono +OXC	Mono +PER	Mono +TPM	Mono +VAL	
	(N = 6746)	(N = 633)	(N = 752)	(N = 1512)	(N = 353)	(N = 420)	(N = 1108)	(N = 1968)	
**Persistence**									
Time on treatment (days)	307.3 ± 92.3	290.1 ± 99.4	314.0 ± 91.7	325.8 ± 78.9	318.4 ± 85.4	322.3 ± 80.3	294.0 ± 97.3	298.4 ± 96.9	<0.0001
Cumulative persistence									
90 days	98.5	97.3	98.0	99.4	98.6	100.0	98.7	98.0	<0.0001
180 days	83.6	79.2	84.7	89.2	87.3	87.6	79.9	80.8	
270 days	73.4	64.9	77.7	81.2	79.0	79.8	66.9	69.9	
365 days	65.5	55.8	70.0	75.9	71.1	73.3	57.4	60.9	
**Adherence**									
MPR	88.9 ± 25.4	82.7 ± 27.6	91.7 ± 23.4	93.5 ± 21.8	91.5 ± 24.7	91.1 ± 23.5	84.3 ± 27.7	88.0 ± 26.4	<0.0001
Adherent (MPR≥80%)	76.8	67.9	81.1	85.0	81.9	81.7	69.2	74.1	<0.0001
**Treatment pattern**									
Changed	41.0	48.3	37.2	31.9	36.3	35.7	48.1	45.1	<0.0001
Switch/stop	81.4	87.6	77.9	72.9	75.8	70.7	86.5	84.7	<0.0001
Augmented	18.6	12.4	22.1	27.1	24.2	29.3	13.5	15.3	0.0292

AED, anti-epileptic drug; Mono, monotherapy; CBZ, carbamazepine; LTG, lamotrigine; LEV, levetiracetam; OXC, oxcarbazepine; PER, perampanel; TPM, topiramate; VAL, valproate; SD, standard deviation; MPR, medication possession ratio.

*P-value* for time on treatment, MPR, adherent, and treatment pattern were derived from the individual chi-square test or Kruskal-Walis test.

*P-value* for cumulative persistence rate was derived from the log-rank test.

Data are presented as mean ± SD or percentages (%).

The mean MPR of the entire study population was 88.9±25.4% and 76.8% of the population was adherent with MPR ≥80% within the 12 months of follow-up period. Similar to the result of persistence, the LTG, LEV, OXC, and PER groups had higher mean MPR and adherent rate than the CBZ, TPM, or VAL groups (Table 2).

### 3.3 Treatment patterns

Among the entire study population, 2,768 (41.0%) patients changed their index AED add-on during the follow-up period, and the rate of change was relatively higher in the CBZ, TPM, and VAL users than those receiving LTG, LEV, OXC, or PER. Among those who changed their index AED add-on, 81.4% switched to a different AED or stopped their index AED add-on therapy, whereas 18.6% augmented their index AED add-on with another AED. The proportion of patients who switched or stopped their index AED add-on was higher in the CBZ, TPM, and VAL groups than in the LTG, LEV, OXC, or PER groups, whereas the proportion of those who augmented their index AED add-on were higher in the LTG, LEV, OXC, and PER groups (Table 2).

### 3.4 Healthcare resource utilization and costs

In the study population, 35.1% and 5.6% experienced at least one all-cause and one epilepsy-related hospitalization during the follow-up period, respectively. The average number of admissions (per person) and length of stay (per admission) were 2.3 and 30.9 days for all-cause and 1.2 and 18.9 days for epilepsy-related hospitalization, respectively. On average, the cost of hospitalization (per person per year; PPPY) was USD 7,851 and 2,924 for all-cause and epilepsy-related cases, respectively (Table 3). The number of admissions, length of stay and total cost for all-cause hospitalization, and the length of stay for epilepsy-related hospitalization significantly differed between the study groups. The PER group showed the lowest HCRU and costs for all-cause hospitalization, and length of stay for epilepsy-related hospitalization. The mean number of outpatient visits (per person) and cost (PPPY) was 25.4 and USD 1,232 for all-cause and 6.2 and USD 287 for epilepsy-related visits, respectively. Significant differences were observed in the numbers of outpatient visits and costs between the groups. The PER group showed the least number of outpatient visits, regardless of the cause, and the lowest all-cause outpatient cost, whereas the epilepsy-related cost was the lowest in the CBZ group (Table 3). During the follow-up period, 27.6% and 3.8% of the entire study population visited an ED at least once for all-cause and epilepsy-related cases, respectively. The mean number of ED visits (per person) and cost (PPPY) was 1.9 and USD 2,658 for all-cause and 1.2 and USD 1,024 for epilepsy-related visit, respectively. The cost of all-cause ED visits was significantly different between the study groups, with the lowest cost observed in the PER group (Table 3).

**Table 3:** All-cause and epilepsy-related healthcare resource utilization (HCRU) and costs.

	Overall	Type of index AED							*p*-value
		Mono +CBZ	Mono +LTG	Mono +LEV	Mono +OXC	Mono +PER	Mono +TPM	Mono +VAL	
	(N = 6746)	(N = 633)	(N = 752)	(N = 1512)	(N = 353)	(N = 420)	(N = 1108)	(N = 1968)	
**All-cause**									
**Hospitalization**									
Patients ≥1 admission	2365 (35.1)	279 (44.1)	220 (29.3)	505 (33.4)	125 (35.4)	108 (25.7)	344 (31.0)	784 (39.8)	<0.0001
Number of admissions	2.3 ± 3.5	2.2 ± 1.9	2.2 ± 2.4	2.1 ± 2.0	2.5 ± 5.3	1.8 ± 1.6	2.2 ± 3.6	2.4 ± 4.6	0.0308
Length of stay	30.9 ± 71.4	43.1 ± 83.8	17.3 ± 44.1	27.8 ± 64.1	15.3 ± 41.6	10.3 ± 26.0	26.5 ± 66.1	38.5 ± 82.7	<0.0001
Annual cost (USD)	7851 ± 13057	8835 ± 11846	6364 ± 11264	9112 ± 18197	5863 ± 9165	4364 ± 7950	6375 ± 9652	8551 ± 12157	<0.0001
**Outpatient visit**									
Patients ≥1 visit	6646 (98.5)	615 (97.2)	749 (99.6)	1491 (98.6)	351 (99.4)	419 (99.8)	1099 (99.2)	1922 (97.7)	<0.0001
Number of visits	25.4 ± 24.2	32.5 ± 29.1	24.6 ± 23.6	21.0 ± 22.4	24.8 ± 27.3	20.1 ± 19.7	27.0 ± 22.3	27.1 ± 24.7	<0.0001
Annual cost (USD)	1232 ± 2341	1463 ± 2490	1192 ± 1620	1074 ± 3127	1317 ± 2481	919 ± 1441	1314 ± 1530	1302 ± 2322	<0.0001
**ED visit**									
Patients ≥1 visit	1861 (27.6)	178 (28.1)	200 (26.6)	431 (28.5)	107 (30.3)	105 (25.0)	273 (24.6)	567 (28.8)	0.1822
Number of visits	1.9 ± 1.8	2.0 ± 1.7	1.9 ± 2.1	1.8 ± 1.6	1.8 ± 1.2	1.5 ± 1.0	1.9 ± 1.8	2.0 ± 1.9	0.2012
Annual cost (USD)	2658 ± 8172	3033 ± 5166	2585 ± 6759	3862 ± 14263	1722 ± 3329	1017 ± 2442	2181 ± 5334	2361 ± 4654	<0.0001
**Epilepsy-related**									
**Hospitalization**									
Patients ≥1 admission	377 (5.6)	15 (2.4)	42 (5.6)	126 (8.3)	29 (8.2)	29 (6.9)	38 (3.4)	98 (5.0)	<0.0001
Number of admissions	1.2 ± 0.6	1.3 ± 0.8	1.3 ± 0.9	1.3 ± 0.7	1.3 ± 0.5	1.3 ± 0.5	1.3 ± 0.6	1.1 ± 0.4	0.4317
Length of stay	18.9 ± 63.4	50.5 ± 91.3	7.0 ± 24.0	15.1 ± 57.5	6.5 ± 10.5	5.4 ± 5.4	22.6 ± 72.7	32.1 ± 88.4	0.0129
Annual cost (USD)	2924 ± 5519	3956 ± 4728	2520 ± 4980	2257 ± 3808	2453 ± 2825	3352 ± 4931	3584 ± 7480	3552 ± 7314	0.7146
**Outpatient visit**									
Patients ≥1 visit	3424 (50.8)	158 (25.0)	435 (57.8)	1090 (72.1)	233 (66.0)	369 (87.9)	406 (36.6)	733 (37.2)	<0.0001
Number of visits	6.2 ± 3.7	6.6 ± 4.5	6.1 ± 3.3	6.1 ± 3.1	6.4 ± 3.9	5.7 ± 4.0	6.2 ± 3.9	6.6 ± 4.1	0.0020
Annual cost (USD)	287 ± 327	225 ± 253	312 ± 332	283 ± 298	343 ± 533	282 ± 378	274 ± 301	285 ± 274	0.0019
**ED visit**									
Patients ≥1 visit	257 (3.8)	8 (1.3)	34 (4.5)	93 (6.2)	21 (5.9)	13 (3.1)	23 (2.1)	65 (3.3)	<0.0001
Number of visits	1.2 ± 0.6	1.1 ± 0.4	1.5 ± 1.1	1.2 ± 0.5	1.2 ± 0.5	1.1 ± 0.3	1.3 ± 0.6	1.2 ± 0.4	0.7399
Annual cost (USD)	1024 ± 1203	1086 ± 1120	1055 ± 1735	1029 ± 1237	1149 ± 1225	565 ± 531	1012 ± 836	1049 ± 1040	0.6506

AED, anti-epileptic drug; Mono, monotherapy; CBZ, carbamazepine; LTG, lamotrigine; LEV, levetiracetam; OXC, oxcarbazepine; PER, perampanel; TPM, topiramate; VAL, valproate; HCRU, healthcare resource utilization; PYs, person-years; SD, standard deviation; USD, U.S. dollar; ED, emergency department.

HCRU and cost were estimated with healthcare users only.

*P-value* for number of admissions, number of visits, length of stay, and cost were derived from individual chi-square test or Kruskal-Walis test.

The cost was converted from South Korean Won (KRW) to USD using the average foreign exchange rate in the year of 2017 (1 US Dollar = 1,178.585 South Korean Won).

Data are presented as mean ± SD or n (%).

The Number of visits, the number of admissions, and annual cost are presented as per person and the length of stay is presented as per admission.

## 4 Discussion

This retrospective, nationwide study compared treatment compliance, treatment patterns, HCRU and costs of AED add-on therapy that was initially added to AED monotherapy in a real-world clinical setting in an Asian population.

In our study, the use of LTG, LEV, OXC, or PER as the first AED add-on to monotherapy was associated with better persistence and fewer changes in AED add-on therapy than CBZ, TPM, or VAL use. Furthermore, patients treated with LTG, LEV, OXC, or PER were less likely to switch or stop their first AED add-on than those on CBZ, TPM, or VAL in real-world clinical settings. These results are consistent with those observed from previous studies even though the study population (i.e., new users or existing users) and AED therapy of interest (i.e., monotherapy or polytherapy) varied across the studies. In a retrospective study that assessed persistence of AED monotherapy in newly diagnosed epilepsy patients, LTG (hazard ratio [HR] 0.72, 95% confidence interval [CI] 0.65–0.81) and OXC (HR 0.78, 95%CI 0.74–0.83) showed a lower risk of non-persistence than CBZ [23]. Another retrospective study conducted in Scotland observed that second-generation AEDs (odds ratio [OR] 1.26, p=0.004) including LTG, LEV, lacosamide (LCS), and zonisamide (ZNA) demonstrated higher persistence rates than first-generation AEDs in new users and previously non-persistent users [24]. Faught et al. also reported, using the US claims data, that the persistence time on the initial AED monotherapy was longer in LTG, LEV, and OXC groups than in gabapentin (GBN), phenytoin (PHT), or VAL groups [25]. Similar results were observed in a pragmatic randomized clinical trial with newly diagnosed or treated epilepsy patients. The SANAD study found that the time to treatment failure of AED monotherapy was significantly better in LTG users than in CBZ (HR 0.78, 95% CI 0.63–0.97) or GBN (HR 0.65, 95% CI 0.52–0.80) users [26]. Similar to the results of persistence, we found that the LTG, LEV, OXC, and PER groups showed higher adherence and adherent with MPR≥80% than the CBZ, TPM, or VAL groups. These findings are consistent with those of a previous observational study. In a German retrospective, population-based study, LTG, LEV, and OXC groups showed higher adherence rates than VAL group [27]. Our findings on treatment persistence and adherence suggest that the use of LTG, LEV, OXC, or PER as the first AED add-on to monotherapy could be beneficial in managing epilepsy compared with that of CBZ, TPM, or VAL. Previous studies have demonstrated that better adherence is associated with improved seizure control, quality of life, and lower risk of morbidity [28,29]. Further, better persistence may indicate clinical benefits related to efficacy, safety, and tolerability. Therefore, several previous studies evaluating AED effectiveness have measured persistence as a primary outcome, and the Commission on Antiepileptic Drugs of the International League Against Epilepsy recommends using persistence in evaluating AED effectiveness [7,26,30].

In our study, the overall persistence and adherence during the 12-month follow-up period was 65.5% and 88.9%, respectively. These results tended to be higher than those observed in previous studies. According to Marshall et al., the persistence rate of existing users with additional AEDs ranged from 11.7% to 54.0% at 365 days [24]. A US study that used claims data to assess adherence to AED therapy reported a mean MPR of 78% and a 60.7% adherent rate (MPR≥80%) [31]. Additionally, Malek et al. reported adherent rate between 50% and 74% [32]. Our study considered as persistent if a patient continued their index AED add-on to monotherapy (i.e., not considered the change or discontinuation of monotherapy), and consequently it was assumed that the mean level of persistence and adherence may be higher in our study.

The association of AED adherence with healthcare utilization and costs has been demonstrated in previous studies [31,33]. A retrospective study using the US insurance claims data reported that non-adherence to AED was significantly associated with increased hospitalization (0.19 additional admission per patient), inpatient days (2.3 additional days), and ER admission (0.225 additional admission per patient) in older patients with epilepsy. This study also found that non-adherent significantly increased hospitalization (USD 872), ER admission (USD 143), and total healthcare costs (USD 2674) PPPY [33]. Another study using the PharMetrics Integrated Outcomes Database identified increased hospitalization and related costs (OR 1.110, p=0.013; USD 1,799 PPPY) and ER admission (OR 1.479, p<0.0001; USD 260 PPPY) in AED non-adherent patients [31]. Similar trends were observed in the present study. The LTG, LEV, OXC, and PER groups, which showed better persistence and adherence, tended to have lower healthcare utilization and costs than the CBZ, TPM, or VAL groups. In particular, the length of stay (per admission) and hospitalization cost (PPPY) were lower in the former groups, regardless of the cause of admission. Furthermore, the PER group in our study showed the lowest healthcare utilization and costs for all-cause cases as well as the lowest length of stay and outpatient visits for epilepsy-related cases. Reduced healthcare utilization in PER-based AED combination therapy was also reported in a recent retrospective study in the US. Using claims data, the study found that PER-based AED combination reduced both all-cause and epilepsy-related hospitalization compared with other AED combination therapies in patients with epilepsy. All-cause clinic/office and outpatient visits were also lower in PER-based combination therapy than in other AED combination therapies [34]. Our findings suggest that using LTG, LEV, OXC, or PER as first add-on therapies could contribute to improving AED persistence and adherence, which could consequently lead to a reduction in healthcare utilization and costs.

This study used national health insurance claims data of the total Korean population, which is approximately 50 million. Therefore, we could completely follow up patients under the routine clinical practice, as determine by the treating physicians and current clinical practice. Consequently, the results of this study may represent the general Korean population in a real-world setting. However, several limitations inherent to most claims database studies should be considered when interpreting the results. First, the diagnosis codes (ICD-10 code) were used to define the target patients and outcomes, including the causes of events. Therefore, there was a possibility of miscoding that might have led to the misclassification of the target population or under- or over-estimation of the outcomes. Second, due to a lack of relevant information, we were not able to identify some demographic and clinical characteristics such as seizure type, frequency, or severity, or socioeconomic status and level of education. These factors might have affected choice of AED add-on therapy as well as treatment persistence and adherence [35,36]. Third, it was not possible to confirm whether the patients actually took the prescribed medications due to the nature of the claims data. Moreover, we were not able to distinguish whether the discontinuation of index AED add-on therapy was due to therapeutic needs such as adverse event or patients’ needs since the information on the reason for discontinuation or changes in medication is not available in the claim data. Finally, the duration of epilepsy, which may impact treatment adherence and persistence, was not considered in this study.

## 5 Conclusion

This nationwide population based real-world study confirms the results of our previous study that the use of LTG, LEV, OXC, or PER as the first add-on to monotherapy in Asian patients with epilepsy may contribute to improved persistence and adherence to add-on therapy than the use of CBZ, TPR, or VAL [17]. This study also showed that the rates of switching and stopping were lower in LTG, LEV, OXC, and PER groups. Furthermore, the study suggests that LTG, LEV, OXC, and PER use could reduce healthcare utilization and costs in real-world clinical settings.

## Ethiscs declaration

This study was reviewed and approved by the Institutional Review Board of Seoul National University Hospital, with the approval number: E−1811-002-982, and HIRA Data Review Committee, with the approval number: M20210226130. The informed consent was waived since the HIRA claims data was structured in de-identified format.

## Funding

This work was supported by 10.13039/501100007033Eisai Korea Inc and Open Access Fees were also funded by Eisai Korea Inc.

## Data availability

The datasets generated and/or analyzed during this study will not be publicly available as the Health Insurance Review and Assessment (10.13039/501100003635HIRA), the data provider, does not provide data without a permission granted for the access to the data. The access to the HIRA's claims data is allowed after obtaining an approval from the HIRA review committee according to the guidance for the use of HIRA claims data.

## CRediT authorship contribution statement

**Min Young Kim:** Writing – review & editing, Writing – original draft, Validation, Methodology, Formal analysis, Data curation, Conceptualization. **Jung-Ae Kim:** Writing – review & editing, Writing – original draft, Validation, Project administration, Methodology, Formal analysis, Data curation, Conceptualization. **Young Eun Lee:** Methodology, Formal analysis, Data curation, Conceptualization. **Sang Kun Lee:** Writing – review & editing, Writing – original draft, Supervision, Methodology, Conceptualization.

## Declaration of competing interest

The authors declare the following financial interests/personal relationships which may be considered as potential competing interests:Sang Kun Lee reports financial support was provided by IQVIA Korea. Jung-Ae Kim reports financial support was provided by Eisai Korea Inc. Young Eun Lee reports financial support was provided by Eisai Korea Inc. Min Young Kim is an employee of Eisai Korea Inc.
