# Supplementary material for: Treatment compliance, treatment patterns, and healthcare utilization in epilepsy patients with first add-on antiepileptic drugs: A nationwide cohort study
Source: Heliyon. 2024 Mar 7;10(6):e27770. doi: 10.1016/j.heliyon.2024.e27770 (PMC10945250; doi:10.1016/j.heliyon.2024.e27770)
Supplement: Multimedia component 1 [file mmc1.docx]

**Title: Treatment compliance, treatment patterns, and healthcare utilization in epilepsy patients with first add-on antiepileptic drugs: a nationwide cohort study**

Min Young Kim^a^, Jung-Ae Kim^b^, Young Eun Lee^b^, Sang Kun Lee^c*^

^a^ Medical, Eisai Korea Inc, 6 Bongeunsa-ro 86-gil, Gangnam-gu, Seoul, Republic of Korea

^b^ Real World Insights, IQVIA Korea, 173 Toegye-ro, Jung-gu, Seoul, Republic of Korea

^c^ Department of Neurology, Seoul National University Hospital, 101 Daehak-ro, Jongno-gu, Seoul, Republic of Korea

***Corresponding author:**

Sang Kun Lee, MD, PhD

Department of Neurology, Seoul National University Hospital

101 Daehak-ro, Jongno-gu, Seoul 03080, Republic of Korea

E-mail: sangkun2923@gmail.com

Phone +82-2-2072-2923/Fax +82-2-3762-7553

**Supplementary materials:**

I. Supplementary Methods

II. Supplementary Tables

I. Supplementary Methods

**Persistence**

The cumulative persistence rates were assessed using the Kaplan-Meier analysis and compared using the log-rank test. The cumulative persistence was further measured according to demographic and clinical characteristics observed during the baseline period.

II. Supplementary Tables

Supplementary Table 1. Relevant codes and operational definition of inclusion and exclusion criteria.

| **Criteria** | **Class** | **Code** | **Operational definition** |
| --- | --- | --- | --- |
| **Epilepsy** |  |  |  |
| Epilepsy | Diagnosis | G40.x, G41.x | ≥1 claim with defined diagnosis code as any diagnosis |
| **AED** |  |  |  |
| **Targeted AED** |  |  |  |
| Carbamazepine | Drug | 123102ATB, 123102ATR, 123103ASY, 123104ATR, 123130ASY | ≥1 claim with defined drug code |
| Lamotrigine | Drug | 181001ATB, 181002ATB, 181003ATB, 181004ATB, 181005ATB |  |
| Levetiracetam | Drug | 488501ATB, 488502ATB, 488503ATB, 488505ATB, 488501ATR, 488505ATR, 488530ALQ, 488504ALQ, 488531ALQ, 488536ALQ |  |
| Oxcarbazepine | Drug | 206301ATB, 206302ATB, 206303ATB, 206304ASS, 206330ASS, 206331ASS |  |
| Perampanel | Drug | 642401ATB, 642402ATB, 642403ATB, 642404ATB, 642405ATB, 642406ATB, 642407ASS |  |
| Topiramate | Drug | 241801ATB, 241803ATB, 241804ATB, 241801ACR, 241802ACR, 241803ACR, 241804ACR, 241803ACH, 241804ACH |  |
| Valproate (Valproic acid, divalproex sodium, sodium valproate, valproate magnesium) | Drug | 147701ATB, 147701ATE, 147701ATR, 147702ATB, 147702ATE, 147702ATR, 147801ACH, 147801ACS, 229701ACR, 229701ATR, 229703ATB, 229705ACR, 229705ATB, 229705ATR, 229706AGR, 229706ATB, 229706ATR, 229707ATR, 229708ASY, 229709AGR, 229710AGR, 229711AGR, 229712AGR, 229733ASY, 229734ASY, 229735ASY, 246901ATB, 246901ATE, 246902ATB, 246902ATE, 246903ATB, 246903ATE, 247001ACS, 247002ACS |  |
| **Non-targeted AED** |  |  |  |
| Clonazepam | Drug | 136401ATB | ≥1 claim with defined drug code |
| Ethosuximide | Drug | 155901ACS |  |
| Gabapentin | Drug | 164201ACH, 164202ACH, 164203ACH, 164204ATB, 164205ATB |  |
| Lacosamide | Drug | 611702ATB, 611703ATB, 611704ATB, 611705ATB |  |
| Phenobarbital | Drug | 211701ATB |  |
| Phenytoin(diphenylhydantoin) | Drug | 146801ACH, 146801ATB |  |
| Pregabalin | Drug | 480401ACH, 480402ACH, 480403ACH, 480404ACH, 480405ACH, 480406ACH, 480401ATB, 480402ATB, 480405ATB, 480406ATB, 480402ATR, 480403ATR, 480407ATR, 480408ATR, 480409ATR |  |
| Primidone | Drug | 217801ATB |  |
| Rufinamide | Drug | 509401ATB, 509402ATB, 509403ATB |  |
| Stiripentol | Drug | 509101ACH, 509101ACS |  |
| Vigabatrin | Drug | 247701ATB |  |
| Zonisamide | Drug | 250601ATB, 250602APD, 250630APD |  |
| Phenobarbital + Phenytoin | Drug | 251600ATB |  |

AED, anti-epileptic drug.

The diagnosis was coded according to the Korea Classification of Disease code modification of the International Classification of Disease-10 code. The drug was coded according to Health Insurance Review and Assessment’s molecule code.

Supplementary Table 2. Relevant codes and operational definitions of healthcare resource utilization (HCRU).

| **Criteria** | **Class** | **Code** | **Operational definition** |
| --- | --- | --- | --- |
| **Hospitalization** |  |  |  |
| Epilepsy-related hospitalization |  |  |  |
| Epilepsy | Diagnosis | G40.xx, G41.xx | ≥1 inpatient claim with defined diagnosis code as primary diagnosis during the follow-up period |
| **Outpatient visit** |  |  |  |
| Epilepsy-related outpatient visit |  |  |  |
| Epilepsy | Diagnosis | G40.xx, G41.xx | ≥1 outpatient claim with defined diagnosis code as primary diagnosis during the follow-up period |
| **Emergency Department (ED) visit** |  |  |  |
| All-cause emergency department visit |  |  |  |
| Management of emergency care | Procedure | AC101, AC103, AC105, V1100, V1200, V1210, V1220, V1300, V1310, V1320, V1400, V1500, V1510, V1520, V1800, V1810, V1820 | ≥1 claim with defined management of emergency care code, emergency clinical consultation fee code, observation of patients with severe emergencies code, or emergency special ICU room code during the follow-up period |
| Emergency clinic consultation fee | Procedure | V2100, V2200, V2300, V2500 |  |
| Observation of patients with severe emergencies | Procedure | V3100, V3101, V3102, V3103, V3104, V3105, V3106, V3108, V3109, V3200, V3201, V3202, V3203, V3204, V3205, V3206, V3208, V3209, V3210, V3211, V3212, V3213, V3214, V3215, V3216, V3218, V3219, V3220, V3221, V3222, V3223, V3224, V3225, V3226, V3228, V3229, V4100, V4101, V4102, V4103, V4104, V4105, V4106, V4108, V4109, V4200, V4201, V4202, V4203, V4204, V4205, V4206, V4208, V4209, V4210, V4211, V4212, V4213, V4214, V4215, V4216, V4218, V4219, V4220, V4221, V4222, V4223, V4224, V4225, V4226, V4228, V4229, V4300, V4301, V4302, V4303, V4304, V4305, V4306, V4308, V4309, V4310, V4311, V4312, V4313, V4314, V4315, V4316, V4318, V4319, V4320, V4321, V4322, V4323, V4324, V4325, V4326, V4328, V4329, V4500, V4501, V4502, V4503, V4504, V4505, V4506, V4508, V4509, V4510, V4511, V4512, V4513, V4514, V4515, V4516, V4518, V4519, V4520, V4521, V4522, V4523, V4524, V4525, V4526, V4528, V4529, V4800, V4801, V4802, V4803, V4804, V4805, V4806, V4808, V4809, V4810, V4811, V4812, V4813, V4814, V4815, V4816, V4818, V4819, V4820, V4821, V4822, V4823, V4824, V4825, V4826, V4828, V4829 |  |
| Emergency special ICU room management fee | Procedure | V5100, V5200, V5210, V5220, V5500, V5510, V5520 |  |
| Epilepsy-related emergency department visit |  |  |  |
| Management of emergency care | Procedure | AC101, AC103, AC105, V1100, V1200, V1210, V1220, V1300, V1310, V1320, V1400, V1500, V1510, V1520, V1800, V1810, V1820 | ≥1 claim with defined diagnosis code as primary diagnosis and management of emergency care code, emergency clinical consultation fee code, observation of patients with severe emergencies code, or emergency special ICU room code during the follow-up period |
| Emergency clinic consultation fee | Procedure | V2100, V2200, V2300, V2500 |  |
| Observation of patients with severe emergencies | Procedure | V3100, V3101, V3102, V3103, V3104, V3105, V3106, V3108, V3109, V3200, V3201, V3202, V3203, V3204, V3205, V3206, V3208, V3209, V3210, V3211, V3212, V3213, V3214, V3215, V3216, V3218, V3219, V3220, V3221, V3222, V3223, V3224, V3225, V3226, V3228, V3229, V4100, V4101, V4102, V4103, V4104, V4105, V4106, V4108, V4109, V4200, V4201, V4202, V4203, V4204, V4205, V4206, V4208, V4209, V4210, V4211, V4212, V4213, V4214, V4215, V4216, V4218, V4219, V4220, V4221, V4222, V4223, V4224, V4225, V4226, V4228, V4229, V4300, V4301, V4302, V4303, V4304, V4305, V4306, V4308, V4309, V4310, V4311, V4312, V4313, V4314, V4315, V4316, V4318, V4319, V4320, V4321, V4322, V4323, V4324, V4325, V4326, V4328, V4329, V4500, V4501, V4502, V4503, V4504, V4505, V4506, V4508, V4509, V4510, V4511, V4512, V4513, V4514, V4515, V4516, V4518, V4519, V4520, V4521, V4522, V4523, V4524, V4525, V4526, V4528, V4529, V4800, V4801, V4802, V4803, V4804, V4805, V4806, V4808, V4809, V4810, V4811, V4812, V4813, V4814, V4815, V4816, V4818, V4819, V4820, V4821, V4822, V4823, V4824, V4825, V4826, V4828, V4829 |  |
| Emergency special ICU room management fee | Procedure | V5100, V5200, V5210, V5220, V5500, V5510, V5520 |  |

ED, emergency department; ICU, intensive care unit.

The procedure was coded according to Health Insurance Review and Assessment reimbursement code.

Supplementary Table 3. Relevant codes and operational definition for type of epilepsy, mCCI, and comorbidities.

| **Baseline characteristics** | **Class** | **Code** | **Operational definition** | |
| --- | --- | --- | --- | --- |
| **Type of epilepsy** |  |  |  | |
| Epilepsy with partial seizure | Diagnosis | G40, G40.0, G40.1, G40.2, G41, G41.2 | ≥1 claim with defined diagnosis codes in any diagnosis at index date Type of epilepsy with high-ranked diagnosis was chosen when ≥2 type of epilepsy were observed at index date | |
| Epilepsy with generalized seizure | Diagnosis | G40.3, G40.4, G40.5, G40.6, G40.7, G41.0, G41.1 |  |  |
| Epilepsy with unspecified seizure | Diagnosis | G40.8, G40.9, G41.8, G41.9 |  |  |
| Unknown |  |  | No claim with defined diagnosis code for partial seizure, generalized seizure, or unspecified seizure as any diagnosis at index date | |
| **mCCI** |  |  |  | |
| Congestive heart failure (2 point) | Diagnosis | I09.9; I11.0, I13.0; I13.2; I25.5; I42.0; I42.5-I42.9; I43.x; I50.x; P29.0 | ≥1 claim with defined diagnosis code as primary-6th diagnosis during baseline period | |
| Dementia (2 point) | Diagnosis | F00.x-F03.x; F05.1; G30.x, G31.1 |  |  |
| Chronic pulmonary disease (1 point) | Diagnosis | I27.8; I27.9; J40.x-J47.x; J60.x-J67.x; J68.4; J70.1; J70.3 |  |  |
| Rheumatologic disease (1 point) | Diagnosis | M05.x; M06.x; M31.5; M32.x-M34.x; M35.1; M35.3; M36.0 |  |  |
| Mild liver disease (2 point) | Diagnosis | B18.x; K70.0-K70.3; K70.9; K71.3-K71.5; K71.7; K73x; K74x; K76.0; K76.2-K76.4; K76.8; K76.9; Z94.4 |  |  |
| Diabetes with chronic complications (1 point) | Diagnosis | E10.2-E10.5; E10.7; E11.2-E11.5; E11.7; E12.2-E12.5; E12.7; E13.2-E13.5; E13.7; E14.2-E14.5; E14.7 |  |  |
| Hemiplegia or paraplegia (2 point) | Diagnosis | G04.1; G11.4; G80.1; G80.2; G81.x; G82.x; G83.0-G83.4; G83.9 |  |  |
| Renal disease (1 point) | Diagnosis | I12.0; I13.1; N03.2-N03.7; N05.2-N05.7; N18.x; N19.x; N25.0; Z49.0-Z49.2; Z94.0; Z99.2 |  |  |
| Any malignancy, including lymphoma and leukemia (2 point) | Diagnosis | C00.x-C26.x; C30.x-C34.x; C37.x-C41.x; C43.x; C45.x-C58.x; C60.x-C76.x; C81.x-C85.x; C88.x; C90.x-C97.x |  |  |
| Moderate or severe liver disease (4 point) | Diagnosis | I85.0; I85.9; I86.4; I98.2; K70.4; K71.1; K72.1; K72.9; K76.5; K76.6; K76.7 |  |  |
| Metastatic solid tumor (6 point) | Diagnosis | C77.x-C80.x |  |  |
| HIV (4 point) | Diagnosis | B20.x-B22.x; B24.x |  |  |
| **Psychiatric comorbidity** |  |  |  | |
| Depression | Diagnosis | F32, F33.0, F33.1, F33.2, F33.3, F33.4, F33.8, F33.9, F34.1, F41.2 | ≥1 claim with defined diagnosis code as primary-6th diagnosis during baseline period | |
| Anxiety disorder | Diagnosis | F40, F41, F42, F43, F44, F45, F48 |  |  |
| Bipolar disorder | Diagnosis | F31 |  |  |
| Schizophrenia | Diagnosis | F20 |  |  |
| **Neurological comorbidity** |  |  |  | |
| Mental retardation | Diagnosis | F70, F71, F72, F73, F78, F79 | ≥1 claim with defined diagnosis code as primary-6th diagnosis during baseline period | |
| **Number of AED** |  |  | |  |
| Carbamazepine | Drug | 123102ATB, 123102ATR, 123103ASY, 123104ATR, 123130ASY | | ≥1 claim with defined drug code |
| Lamotrigine | Drug | 181001ATB, 181002ATB, 181003ATB, 181004ATB, 181005ATB | |  |
| Levetiracetam | Drug | 488501ATB, 488502ATB, 488503ATB, 488505ATB, 488501ATR, 488505ATR, 488530ALQ, 488504ALQ, 488531ALQ, 488536ALQ | |  |
| Oxcarbazepine | Drug | 206301ATB, 206302ATB, 206303ATB, 206304ASS, 206330ASS, 206331ASS | |  |
| Perampanel | Drug | 642401ATB, 642402ATB, 642403ATB, 642404ATB, 642405ATB, 642406ATB, 642407ASS | |  |
| Topiramate | Drug | 241801ATB, 241803ATB, 241804ATB, 241801ACR, 241802ACR, 241803ACR, 241804ACR, 241803ACH, 241804ACH | |  |
| Valproate (Valproic acid, divalproex sodium, sodium valproate, valproate magnesium) | Drug | 147701ATB, 147701ATE, 147701ATR, 147702ATB, 147702ATE, 147702ATR, 147801ACH, 147801ACS, 229701ACR, 229701ATR, 229703ATB, 229705ACR, 229705ATB, 229705ATR, 229706AGR, 229706ATB, 229706ATR, 229707ATR, 229708ASY, 229709AGR, 229710AGR, 229711AGR, 229712AGR, 229733ASY, 229734ASY, 229735ASY, 246901ATB, 246901ATE, 246902ATB, 246902ATE, 246903ATB, 246903ATE, 247001ACS, 247002ACS | |  |
| Clonazepam | Drug | 136401ATB | |  |
| Ethosuximide | Drug | 155901ACS | |  |
| Gabapentin | Drug | 164201ACH, 164202ACH, 164203ACH, 164204ATB, 164205ATB | |  |
| Lacosamide | Drug | 611702ATB, 611703ATB, 611704ATB, 611705ATB | |  |
| Phenobarbital | Drug | 211701ATB | |  |
| Phenytoin(diphenylhydantoin) | Drug | 146801ACH, 146801ATB | |  |
| Pregabalin | Drug | 480401ACH, 480402ACH, 480403ACH, 480404ACH, 480405ACH, 480406ACH, 480401ATB, 480402ATB, 480405ATB, 480406ATB, 480402ATR, 480403ATR, 480407ATR, 480408ATR, 480409ATR | |  |
| Primidone | Drug | 217801ATB | |  |
| Rufinamide | Drug | 509401ATB, 509402ATB, 509403ATB | |  |
| Stiripentol | Drug | 509101ACH, 509101ACS | |  |
| Vigabatrin | Drug | 247701ATB | |  |
| Zonisamide | Drug | 250601ATB, 250602APD, 250630APD | |  |
| Phenobarbital + Phenytoin | Drug | 251600ATB | |  |

mCCI, modified Charlson Comorbidity Index; HIV, human immunodeficiency virus.

The diagnosis was coded according to the Korea Classification of Disease code modification of the International Classification of Disease-10 code.

Supplementary Table 4. Persistence according to baseline characteristics of study population.

| **Time on treatment**  **(days,** **mean±[SD])** | | **Overall** | **Type of index AED** | | | | | | | ***p*-value** |
| --- | --- | --- | --- | --- | --- | --- | --- | --- | --- | --- |
|  |  |  | **Mono +CBZ** | **Mono +LTG** | **Mono +LEV** | **Mono +OXC** | **Mono +PER** | **Mono +TPM** | **Mono +VAL** |  |
|  |  | **(N=6,746)** | **(N=633)** | **(N=752)** | **(N=1,512)** | **(N=353)** | **(N=420)** | **(N=1,108)** | **(N=1,968)** |  |
|  | |  |  |  |  |  |  |  |  |  |
| **Age (years)** | |  |  |  |  |  |  |  |  |  |
|  | 12 to <65 | 311.5 ± 89.9 | 294.7 ± 99.5 | 316.8 ± 89.2 | 330.4 ± 74.8 | 319.2 ± 84.3 | 323.2 ± 79.3 | 294.2 ± 96.9 | 304.1 ± 94.5 | <.0001 |
|  | 65+ | 290.9 ± 99.4 | 282.1 ± 98.9 | 295.2 ± 106.0 | 305.3 ± 92.8 | 315.1 ± 90.7 | 308.8 ± 95.0 | 292.6 ± 99.5 | 281.1 ± 101.9 | 0.0073 |
| **Sex** | |  |  |  |  |  |  |  |  |  |
|  | Male | 312.5 ± 88.9 | 296.2 ± 98.2 | 314.7 ± 90.6 | 327.6 ± 77.0 | 329.5 ± 74.7 | 325.5 ± 77.8 | 300.5 ± 95.3 | 305.2 ± 92.9 | <.0001 |
|  | Female | 301.7 ± 95.4 | 283.6 ± 100.4 | 313.4 ± 92.7 | 323.7 ± 81.2 | 304.6 ± 95.7 | 318.5 ± 83.1 | 288.5 ± 98.6 | 289.7 ± 101.2 | <.0001 |
| **Insurance type** | |  |  |  |  |  |  |  |  |  |
|  | Medical aid | 300.4 ± 97.7 | 295.1 ± 102.6 | 303.8 ± 101.5 | 320.8 ± 84.2 | 289.1 ± 101.7 | 317.5 ± 82.7 | 289.7 ± 100.1 | 297.6 ± 99.1 | 0.0219 |
|  | Health insurance | 309.0 ± 90.8 | 288.0 ± 98.1 | 315.8 ± 89.9 | 326.6 ± 78.1 | 322.5 ± 82.3 | 322.8 ± 80.1 | 295.1 ± 96.5 | 298.6 ± 96.2 | <.0001 |
| **mCCI (score)** | |  |  |  |  |  |  |  |  |  |
|  | 0 | 316.4 ± 87.2 | 291.9 ± 101.5 | 320.6 ± 86.6 | 334.6 ± 69.0 | 330.6 ± 76.2 | 324.2 ± 79.6 | 300.5 ± 94.3 | 309.6 ± 93.4 | <.0001 |
|  | 1 | 306.1 ± 93.3 | 292.5 ± 100.3 | 310.0 ± 97.8 | 334.7 ± 70.9 | 300.7 ± 98.1 | 302.8 ± 92.5 | 287.2 ± 100.2 | 300.9 ± 94.2 | <.0001 |
|  | 2 | 300.9 ± 94.8 | 294.6 ± 97.3 | 300.2 ± 98.1 | 312.8 ± 89.3 | 317.8 ± 85.4 | 325.6 ± 79.3 | 294.1 ± 98.3 | 292.2 ± 97.4 | 0.0259 |
|  | 3 | 295.7 ± 97.1 | 290.7 ± 100.0 | 311.5 ± 93.0 | 324.1 ± 82.0 | 304.6 ± 96.2 | 324.1 ± 70.5 | 286.9 ± 97.2 | 274.0 ± 103.6 | 0.0003 |
|  | 4 + | 291.0 ± 98.9 | 280.7 ± 97.7 | 305.1 ± 98.4 | 298.3 ± 98.3 | 299.4 ± 93.2 | 336.5 ± 63.5 | 278.6 ± 104.3 | 287.9 ± 99.8 | 0.1015 |
| **Psychiatric comorbidity** | |  |  |  |  |  |  |  |  |  |
|  | Any psychiatric comorbidity | 292.1 ± 99.2 | 283.8 ± 101.3 | 295.6 ± 102.2 | 317.9 ± 86.0 | 313.5 ± 88.0 | 306.7 ± 90.0 | 282.4 ± 100.9 | 286.5 ± 100.9 | <.0001 |
|  | Depression | 286.3 ± 101.1 | 283.2 ± 99.1 | 288.4 ± 107.0 | 309.3 ± 92.9 | 311.2 ± 87.9 | 298.8 ± 96.8 | 277.1 ± 100.8 | 282.4 ± 102.5 | 0.0008 |
|  | Anxiety disorders | 290.8 ± 99.8 | 286.6 ± 99.3 | 289.8 ± 105.2 | 319.4 ± 84.4 | 317.3 ± 83.8 | 307.5 ± 90.7 | 281.2 ± 100.8 | 284.1 ± 102.6 | <.0001 |
|  | Bipolar disorder | 279.9 ± 103.3 | 267.1 ± 104.9 | 285.1 ± 107.7 | 290.4 ± 106.1 | 322.3 ± 83.6 | 343.6 ± 64.1 | 269.0 ± 102.6 | 281.9 ± 101.5 | 0.0260 |
|  | Schizophrenia | 284.4 ± 103.9 | 274.6 ± 109.3 | 286.0 ± 113.3 | 302.3 ± 103.0 | 317.8 ± 82.8 | 118.0 ± 0 | 280.0 ± 100.9 | 286.9 ± 102.5 | 0.3887 |
| **Neurological comorbidity** | |  |  |  |  |  |  |  |  |  |
|  | Mental retardation | 309.6 ± 92.7 | 307.4 ± 99.7 | 329.6 ± 83.6 | 328.5 ± 79.4 | 266.2 ± 108.9 | 324.3 ± 74.2 | 273.8 ± 109.1 | 323.7 ± 78.9 | 0.0004 |
| **Hospital type** | |  |  |  |  |  |  |  |  |  |
|  | Clinic | 275.9 ± 108.5 | 276.1 ± 107.9 | 255.3 ± 124.6 | 326.4 ± 77.2 | 278.7 ± 120.3 | 277.1 ± 113.5 | 262.3 ± 111.8 | 272.9 ± 107.6 | 0.0019 |
|  | Hospital | 283.1 ± 105.1 | 276.2 ± 111.7 | 275.0 ± 112.1 | 304.6 ± 102.7 | 210.3 ± 122.4 | 256.5 ± 153.4 | 275.9 ± 105.5 | 286.7 ± 101.1 | 0.0950 |
|  | General hospital | 305.0 ± 92.8 | 285.6 ± 96.3 | 315.1 ± 89.2 | 320.6 ± 84.0 | 315.3 ± 84.9 | 326.7 ± 72.0 | 292.1 ± 98.3 | 297.4 ± 97.3 | <.0001 |
|  | Tertiary hospital | 323.0 ± 79.8 | 314.1 ± 85.1 | 324.7 ± 82.3 | 331.6 ± 71.9 | 332.3 ± 71.4 | 322.5 ± 81.1 | 312.1 ± 83.7 | 318.6 ± 83.7 | 0.0005 |
| **Number of AEDs** | |  |  |  |  |  |  |  |  |  |
|  | 1 | 310.4 ± 90.3 | 286.6 ± 102.8 | 304.9 ± 96.2 | 328.7 ± 76.6 | 319.7 ± 84.8 | 321.1 ± 81.6 | 299.3 ± 95.3 | 305.7 ± 92.4 | <.0001 |
|  | 2+ | 302.6 ± 94.9 | 294.0 ± 95.6 | 321.8 ± 87.0 | 320.0 ± 83.1 | 316.9 ± 86.5 | 325.2 ± 77.3 | 284.0 ± 100.2 | 288.1 ± 102.1 | <.0001 |
| **Types of epilepsy** | |  |  |  |  |  |  |  |  |  |
|  | Epilepsy with partial seizure | 312.6 ± 90.2 | 294.2 ± 95.5 | 321.2 ± 90.2 | 324.8 ± 82.3 | 321.3 ± 90.8 | 323.8 ± 79.1 | 298.4 ± 97.0 | 304.9 ± 93.2 | 0.0015 |
|  | Epilepsy with generalized seizure | 310.9 ± 91.8 | 297.3 ± 102.6 | 322.6 ± 82.1 | 334.5 ± 68.3 | 290.6 ± 101.9 | 289.1 ± 102.2 | 283.0 ± 110.1 | 310.5 ± 91.6 | 0.0008 |
|  | Epilepsy with unspecified seizure | 309.1 ± 90.8 | 285.5 ± 102.7 | 318.8 ± 87.4 | 325.7 ± 78.4 | 321.1 ± 80.8 | 323.4 ± 79.4 | 300.0 ± 92.1 | 298.5 ± 97.6 | <.0001 |
|  | Unknown | 286.3 ± 100.6 | 296.8 ± 91.4 | 275.6 ± 110.6 | 316.4 ± 87.6 | 309.3 ± 87.2 | 365.0 ± 0.0 | 271.6 ± 106.2 | 283.0 ± 100.1 | 0.0098 |

AED, anti-epileptic drug; Mono, monotherapy; CBZ, carbamazepine; LTG, lamotrigine; LEV, levetiracetam; OXC, oxcarbazepine; PER, perampanel; TPM, topiramate; VAL, valproate; SD, standard deviation; mCCI, modified Charlson Comorbidity Index.

*P-value* was derived from individual ANOVA test.

Demographic and socioeconomic characteristics such as age, sex, insurance type, hospital type, and type of epilepsy were measured at the index date.

Comorbidities such as mCCI score, psychiatric comorbidities, neurological comorbidities, and number of AEDs were measured during the baseline period (1 year before the index date).

Data are presented as mean±SD.
